# Investigation of the Occurrence of Cyanotoxins in Lake Karaoun (Lebanon) by Mass Spectrometry, Bioassays and Molecular Methods

**DOI:** 10.3390/toxins13100716

**Published:** 2021-10-10

**Authors:** Noura Alice Hammoud, Sevasti-Kiriaki Zervou, Triantafyllos Kaloudis, Christophoros Christophoridis, Aikaterina Paraskevopoulou, Theodoros M. Triantis, Kamal Slim, Joanna Szpunar, Ali Fadel, Ryszard Lobinski, Anastasia Hiskia

**Affiliations:** 1 National Council for Scientific Research (CNRS), P.O. Box 11-8281, Riad El Solh, Beirut 1107 2260, Lebanon; n.hammoud@univ-pau.fr (N.A.H.); k.slim@laec-cnrs.gov.lb (K.S.); afadel@cnrs.edu.lb (A.F.); 2 Laboratory of Photo-Catalytic Processes and Environmental Chemistry, Institute of Nanoscience and Nanotechnology, National Center for Scientific Research “Demokritos”, Patr. Grigoriou E’ & 27 Neapoleos Str., Agia Paraskevi, 15341 Athens, Greece; s.zervou@inn.demokritos.gr (S.-K.Z.); t.kaloudis@inn.demokritos.gr (T.K.); c.christoforidis@inn.demokritos.gr (C.C.); k.paraskevopoulou@inn.demokritos.gr (A.P.); t.triantis@inn.demokritos.gr (T.M.T.); 3 Institut des Sciences Analytiques et de Physico-Chimie pour l’Environnement et les Matériaux, Université de Pau et des Pays de l’Adour, E2S UPPA, CNRS, IPREM UMR 5254, Hélioparc, 64053 Pau, France; joanna.szpunar@univ-pau.fr (J.S.); ryszard.lobinski@univ-pau.fr (R.L.); 4 Department of Water Quality Control, Athens Water Supply and Sewerage Company (EYDAP SA), 156 Oropou Str., 11146 Athens, Greece; 5 Chemical Engineering Department, National Technical University, Iroon Politechniou 9, Zografou, 15780 Athens, Greece; 6 Chair of Analytical Chemistry, Warsaw University of Technology, Noakowskiego 3, 00-664 Warsaw, Poland

**Keywords:** cyanotoxins (CTs), microcystins (MCs), volatile organic compounds (VOCs), taste and odor (T&O) compounds, SPE-LC-MS/MS, HS-SPME-GC/MS

## Abstract

Lake Karaoun is the largest artificial lake in Lebanon and serves multiple purposes. Recently, intensive cyanobacterial blooms have been reported in the lake, raising safety and aesthetic concerns related to the presence of cyanotoxins and cyanobacterial taste and odor (T&O) compounds, respectively. Here, we communicate for the first time results from a recent investigation by LC-MS/MS covering multiple cyanotoxins (microcystins (MCs), anatoxin-a, cylindrospermopsin, nodularin) in water and fish collected between 2019 and 2020. Eleven MCs were identified reaching concentrations of 211 and 199 μg/L for MC-LR and MC-YR, respectively. Cylindrospermopsin, anatoxin-a and nodularin were not detected. The determination of the total MCs was also carried out by ELISA and Protein Phosphatase Inhibition Assay yielding comparable results. Molecular detection of cyanobacteria (16S rRNA) and biosynthetic genes of toxins were carried out by qPCR. Untargeted screening analysis by GC-MS showed the presence of T&O compounds, such as β-cyclocitral, β-ionone, nonanal and dimethylsulfides that contribute to unpleasant odors in water. The determination of volatile organic compounds (VOCs) showed the presence of anthropogenic pollutants, mostly dichloromethane and toluene. The findings are important to develop future monitoring schemes in order to assess the risks from cyanobacterial blooms with regard to the lake’s ecosystem and its uses.

## 1. Introduction

Cyanobacteria are photosynthetic microorganisms commonly found in surface waters. They can produce a large variety of secondary metabolites, including toxic compounds, known as cyanotoxins (CTs). CTs have various chemical structures and modes of toxicity (Figure S1). Microcystins (MCs) [1] and Nodularin (NOD) [2] are cyclic peptides, both containing the unique L-amino acid Adda ((2S,3S,8S,9S)-3-amino-9-methoxy-2,6,8-trimethyl-10-phenyldeca-4,6-dienoicacid), which is responsible for their hepatotoxic activity [3,4]. Cylindrospermopsin (CYN) is an alkaloid cyanotoxin with cytotoxic, dermatotoxic, hepatotoxic and possibly carcinogenic potency [5]. The alkaloid Anatoxin-a (ATX), also known as “Very Fast Death Factor”, is a bicyclic secondary amine (2-acetyl-9-azabicyclo(1,4,2)non-2-ene) with acute neurotoxicity [6]. Among CTs, MCs is the most frequently reported class [7,8].

Under favorable environmental conditions of temperature, presence of nutrients and light, cyanobacteria can proliferate excessively and form blooms [9]. The presence of CTs in toxic cyanobacterial blooms poses a significant risk to aquatic ecosystems and drinking water sources, while it has been associated with lethal poisonings of wild animals, livestock and humans [10]. The World Health Organization (WHO) established guideline values for MCs, CYN and ATX for drinking-water (lifetime, short-term or acute) and recreational exposure and published guides to hazard identification and management of risks posed by cyanobacteria and their toxins at each step of the water-use system [11].

Lake Karaoun (Qaraoun) is the largest water body in Lebanon and has multiple uses. The reservoir was created in 1959 and is intended for irrigation, hydropower, and recreational activities, as well as an anticipated drinking water supply to the capital, Beirut [12]. However, nutrient enrichment due to the excessive use of fertilizers in the lake’s watershed, untreated sewage and industrial waste water runoffs, are possibly contributing to the degradation of the reservoir’s water quality [13]. In 2009, the cyanobacteria *Aphanizomenon ovalisporum* and *Microcystis aeruginosa* were first reported and were seen to dominate the lake’s phytoplankton. Their proliferation was attributed to eutrophication and climate change resulting in the extension of dry periods and the rise in water temperature, as well as the pollution from multiple-industrial and agricultural sources [14]. In recent years, the blooms have become more intense, forming thick scums and emitting unpleasant odors in the area.

There is limited information on toxic cyanobacterial blooms in Lake Karaoum [14,15,16], with a previous study reporting the presence of CYN produced by *Aphanizomenon ovalisporum,* however, not confirmed by mass spectrometry [17]. There are no published data regarding the presence of MCs in the reservoir, especially during incidents of cyanobacterial blooms. Due to the multiple uses of the reservoir, including the supply of drinking water, there is an urgent need to definitively assess and confirm the presence of CTs in Lake Karaoun, in order to enable the design of future monitoring programs and to develop related management strategies.

In addition to CTs, cyanobacteria produce a plethora of volatile secondary metabolites, with several of them having a strong taste and odor (T&O). This can make reservoirs unacceptable as drinking water sources due to consumer complaints and can negatively impact recreational activities and tourism [18,19]. Indeed, concerning Lake Karaoun, there has been circumstantial reporting of bad smells, but the presence of T&O compounds has never been investigated and it is not clear if the origin of T&O is from cyanobacteria or from industrial pollution. T&O compounds can be present at very low concentrations and although they are generally not toxic, they are responsible for the unpleasant smell of drinking water. Their removal may require re-planning and additional investments in water treatment [20].

The primary aim of this communication is to report for the first time conclusive results of the presence of CTs (MCs, CYN, ATX) in Lake Karaoun, using mass spectrometric analytical techniques, in addition to bioassays and molecular methods. A further aim is to investigate the presence of T&O compounds, in order to understand whether they originate from cyanobacterial metabolism or from anthropogenic pollution. To meet these objectives, a set of complementary methods were used to produce reliable results for a variety of cyanotoxins and T&O compounds. Since Middle-Eastern lakes (with the exception of Lake Kinneret [21]) are under-studied in regard to toxic cyanobacteria, the results presented give important information on the presence of CTs and T&O compounds in the area, enabling future monitoring programs and management strategies for Lake Karaoun.

## 2. Results

### 2.1. Water Quality and Diversity of Cyanobacteria in Lake Karaoun

**Figure 1:**
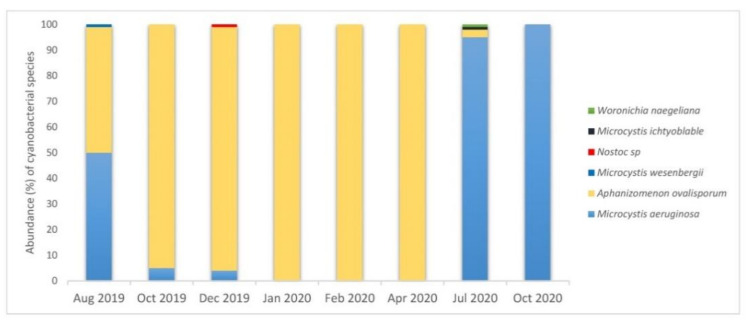
Abundance (%) of cyanobacterial species in Lake Karaoun per sampling month (August 2019–October 2020).

**Figure 2:**
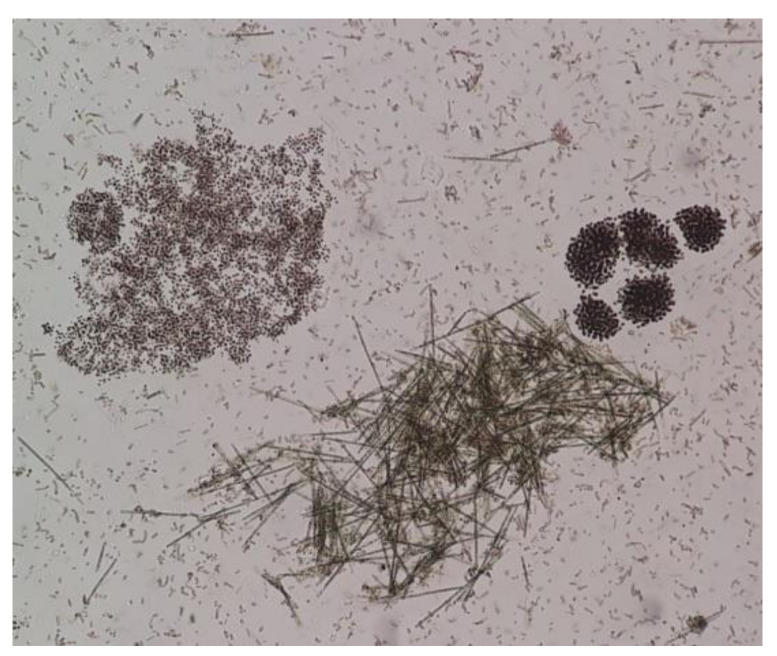
Dominant cyanobacteria species in Lake Karaoun: *Microcystis aeruginosa* and *Aphanizomenon ovalisporum.*

During the period of field campaigns (2019–2020), the diversity of cyanobacteria in the reservoir was limited (Figure 1). *Aphanizomenon ovalisporum* was the dominant species (>95%) from October 2019 to April 2020, while *Microcystis aeruginosa* was the most abundant on July and October 2020. In August 2019, both *Microcystis aeruginosa* and *Aphanizomenon ovalisporum* were equally present with abundances of 50% and 49%, respectively. In addition, in July of the same year, *Microcystis ichtyoblable* and *Woronichia naegeliana* were identified in very low abundances, of 1% each. A microscope image of the dominant cyanobacterial species, i.e., *Microcystis aeruginosa* and *Aphanizomenon ovalisporum* is shown in Figure 2.

Physico-chemical parameters of the lake’s water were also monitored during the sampling period by the National Litani River Authority, which is in charge of the river management (Table S5). In most of the sampling dates, concentrations of nitrate and phosphate exhibited elevated values up to 16.72, 0.28 and 0.16 expressed as mg/L of NO_3_-N, NO_2_-N and PO_4_-P, respectively. This is in agreement with the assessment of the ecological status of the Karaoun reservoir by Fadel et al., classifying the lake as hypereutrophic, with low phytoplankton biodiversity and regular blooms of toxic cyanobacteria [15,16]. Degradation of the reservoir’s water quality is considered to be mainly due to the significant loads of untreated sewage water and the discarding of agricultural and livestock remnants into the river stream.

### 2.2. Occurrence of Cyanotoxins (CTs) in Lake Karaoun

Occurrence of CTs in samples from Lake Karaoun was assessed by liquid chromatography tandem mass spectrometry (LC-MS/MS), complemented by ELISA, Protein Phosphatase Inhibition Assay (PPIA) and molecular detection of cyanobacteria and toxin genes by qPCR. 

#### 2.2.1. LC-MS/MS Analysis of Water Samples

Water samples were analyzed by LC-MS/MS for detection and quantitative determination of extracellular and intracellular CTs, i.e., ATX, CYN, NOD and 12 MC variants (Table 1). Cyanotoxins were found in 50% of samples (8 out of 16), extending over 2019 and 2020. CYN, ATX and NOD were not detected in any of the samples. On the other hand, 11 out of 12 MC variants i.e., dmMC-RR, MC-RR, MC-YR, dmMC-LR, MC-LR, MC-HilR, MC-WR, MC-LA, MC-LY, MC-LW and MC-LF were detected in at least one sample, while MC-HtyR was not detected in any samples. The samples with the largest diversity of MCs were sample 2 (S2-October 19, 10 variants), sample 16 (S3-October 20, 9 variants) and sample 4 (S1-December 19, 5 variants). MC-RR was the most frequently detected toxin (8 out of 16 samples). The sample with the highest concentrations of MCs was sample 16 (S3-October 20), where total MC-LR (sum of extracellular and intracellular fractions) reached 211 μg/L and MC-YR 199 μg/L. In this sample, the dissolved (extracellular) amounts of MC-LR and MC-YR were roughly 100-fold of the intracellular amounts, in contrast to samples 2 (S2-October 19) and 4 (S1-December 19), where MC-LR, MC-RR and MC-YR are found mostly as intracellular. The high proportion of extracellular MCs in sample 16 can possibly be attributed to a decaying phase of the bloom, with disruption of cyanobacteria cells and release of MCs into water. An indicative MRM chromatogram (sample 2, S2-02/10/2019—intracellular fraction) is presented in Figure S2. 

#### 2.2.2. ELISA and PPIA Analysis of Water Samples

Since LC-MS/MS analysis targeted only a limited number of specific MC variants (12), assessment of the presence of MCs was complemented with ELISA and Protein Phosphatase Inhibition Assay (PPIA), in order to estimate total MCs concentration based on structural (ELISA) and functional (PPIA) similarities. Samples were analyzed for extracellular (dissolved) and intracellular MCs, similarly to LC-MS/MS. Results of ELISA and PPIA, together with the sum of MCs determined by LC-MS/MS, for comparison, are presented in Table 2. In general, samples in which either MCs were not detected by LC-MS/MS or their concentrations were lower than the LODs of ELISA/PPIA (0.10/0.25 μg/L), were negative by ELISA and PPIA with the exception of samples 8 (S5–17/2/2020), 11 (S1-15/04/2020), 12 (S2-22/04/2020) and 13 (S4-22/04/2020). Especially, in the latter sample, MCs were detected only by ELISA/PPIA as extracellular, in comparable concentrations (2.30/2.32 μg/L eq. MC-LR). MCs were found mostly as extracellular in sample 16 (S3-9/10/2020) by ELISA and PPIA, in agreement to LC-MS/MS, although in this sample ELISA and PPIA gave about 55% and 32% lower concentrations than LC-MS/MS.

#### 2.2.3. Molecular Detection of Cyanobacteria and Cyanotoxin Genes with qPCR

Molecular detection of cyanobacteria (16S rRNA) and biosynthetic genes of MCs, NOD, CYN, and Saxitoxins (STX) was carried out by qPCR. All samples were found positive for the presence of cyanobacteria. Samples 2 (S2—02/10/2019), 4 (S1—22/12/2019) and 16 (S3—09/10/2020) were found positive for MC producing genes (*mcyE*), with sample 4 being the most abundant in both cyanobacteria and MC genes among all samples. Genes associated with production of CYN (*cyrA*) and STX (*stxA*) were not detected in any of the samples. Results (gene copies per ml of sample) are presented in Table 3. 

#### 2.2.4. LC-MS/MS Analysis of MCs in Fish Samples 

Fish (*Cyprinus caprio*) flesh and liver samples were also analyzed for 12 MC variants by LC-MS/MS, using an in-house method. MCs were not detected in the analyzed samples. An indicative LC-MS/MS MRM chromatogram of a fish liver sample along with the chromatogram of a multi-toxin standard (Figure S3), shows the absence of MCs. 

### 2.3. Taste and Odor (T&O) and Volatile Organic Compounds (VOCs)

**Figure 3:**
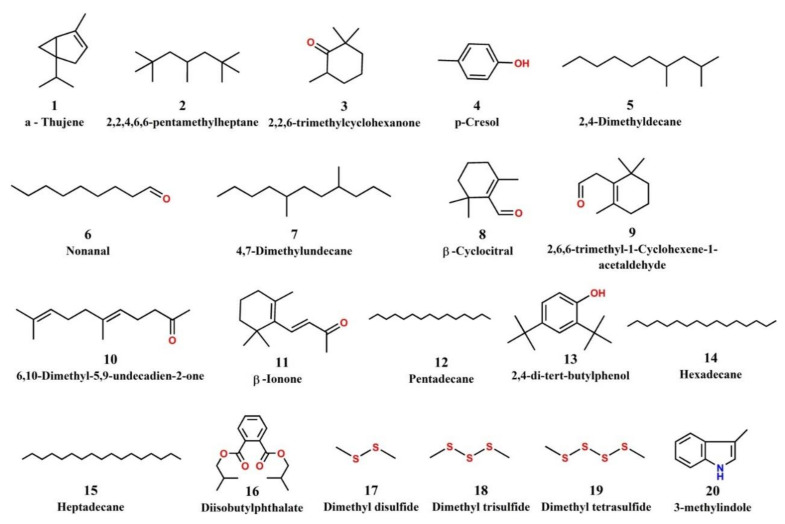
Chemical structures of the volatile compounds identified.

Untargeted screening of volatile compounds by Headspace Solid Phase Microextraction coupled to Gas Chromatography-Mass Spectrometry (HS-SPME-GC-MS) led to the detection and identification of 20 compounds belonging to the following chemical groups: terpenes/terpenoids, hydrocarbons, aldehydes/ketones, phenols, phthalates, alkyl sulfides and indoles (Table 4). The chemical structures of the identified volatile compounds are presented in Figure 3. Two typical cyanobacterial terpenoids T&O compounds, β-cyclocitral (tobacco/woody odor) and β-ionone (floral odor), were detected in samples 2, 4 and 16 and 4, 12 and 16, respectively (Table S2). Nonanal, a known cyanobacterial/algal aldehyde with a fishy odor [24], was detected in 9 samples. Dimethyl disulfide, dimethyl trisulfide and dimethyl tetrasulfide, which are alkyl sulfides associated with algae/cyanobacteria were detected in sample 4, which had a strong swampy/septic odor. Samples 2 and 4 also contained 3-methylindole (skatole), a fecal compound with a characteristic odor. A number of hydrocarbons were also detected, with 2,2,4,6,6-pentamethyl heptane being the most common, as it was found in all samples. Industrial pollutants 2,4-di-tert-butylphenol, diisobutyl phthalate and p-cresol were detected in samples 10, 4 and 2, respectively. Results of untargeted HS-SPME-GC/MS screening per sample are given in Table S2.

Quantitative determination of 36 VOCs (given in 5.8.2) was carried out by HS-SPME-GC/MS. The targeted compounds are typical industrial pollutants of anthropogenic origin. Concentrations of VOCs which were detected in at least one sample are presented in Table 5. All samples were found to contain dichloromethane (25.1–34.0 μg/L). Toluene was present in 12 samples at concentrations up to 0.81 μg/L. Trichloromethane (chloroform) was detected at low concentrations in samples 11 and 16 (0.40 and 0.50 μg/L, respectively). Traces (<0.25 μg/L) of trichlorobenzenes were detected in sample 6, and traces of 1,2 dichloropropane, benzene, xylenes and styrene in sample 16.

## 3. Discussion

A complementary set of analytical methods was used to investigate and confirm the occurrence of cyanotoxins in Lake Karaoun for the first time. Unambiguous determination of 12 MC congeners, NOD, ATX and CYN was carried out by LC-MS/MS, complemented by ELISA and PPIA for MCs and qPCR for MC, CYN and STX producing genes. Results confirm the presence of several congeners (variants) of microcystins in the lake at elevated concentrations during October 2019 and 2020. These early findings give important information on the occurrence, variants and levels of occurring CTs, and they can be used for the establishment of reliable future monitoring programs to support management of cyanobacteria, cyanotoxins and T&O compounds in the context of the multiple uses of the lake.

LC-MS/MS analysis confirmed the presence of dmMC-RR, MC-RR, MC-YR, dmMC-LR, MC-LR, MC-HilR, MC-WR, MC-LA, MC-LY, MC-LW, and MC-LF, with concentrations at 146.8, 25.8 and 429.3 μg/L in October 2019, December 2019 and October 2020, respectively, expressed as the sum of extra and intracellular fractions. MC-LR was the most abundant MC congener followed by MC-YR and MC-RR. CYN, ATX and NOD were not detected in any of the samples. 

Results from the analysis of water samples using ELISA and PPIA for total MCs concentration were in general agreement with those obtained from LC-MS/MS (Table 2). In samples 11, 12 and 13, the higher values for extracellular fraction obtained by ELISA and PPIA may indicate the presence of MC congeners other than those that were targeted by the LC-MS/MS method used in the study. This limitation, caused by the unavailability of commercial MC reference standards is well known in the literature, and it underscores the need for complementary quantitative analysis for total MCs (e.g., ELISA, PPIA), or for the development of non-targeted methods based on high-resolution mass spectrometry and related mass-spectral databases [25]. However, discrepancies between ELISA/PPIA and LC-MS/MS could also be attributed to the well-documented weaknesses of ELISA/PPIA. In particular, the shortcomings of ELISA are its high susceptibility to matrix effects, the variable cross-reactivity of different MC congeners and the response to degradation and/or transformation products of MCs that can result in overestimations [26]. In addition, PPIA based on the assessment of enzyme activity indicating the overall toxicity, does not have the same sensitivity to the different MCs and may also interfere with other unknown compounds present in the sample, thus resulting in under- or over-estimation of the concentration of toxins [27]. Furthermore, ELISA/PPIA lack the specificity of LC-MS/MS, as they respond to structurally/functionally similar molecules in total, and not to specific MC congeners. ELISA/PPIA are useful, quick, easy approaches for the screening of surface waters for MCs, especially where advanced laboratory infrastructure (e.g., LC-MS/MS) is not easily available. However, they must be considered as quantitative screening techniques for the detection of cyanotoxins [28,29].

Molecular analysis (qPCR) confirmed the presence of cyanobacteria (16s rRNA gene) in all the water samples collected. The gene *mcyE* which is associated with the production of MCs was found in 3 samples, i.e., samples 2 (S2, October 2019), 4 (S1, December 2019 and 16 (S3, October 2020), where the presence of MCs was also confirmed by LC-MS/MS, ELISA and PPIA. The gene responsible for the production of CYN (*cyrA*) was not detected in any of the samples (Table 3). This is in agreement with LC-MS/MS analysis where CYN was also not detected (Table 1).

Although significant concentrations of target MCs were detected in water samples, MCs were not detected in fish samples (flesh or liver). Previous studies also referred to cases where very low concentrations or total absence of MCs were reported in fish tissues collected during toxic cyanobacterial blooms [30,31]. It was shown that concentrations of MCs in fish tissues is strongly affected by local bloom conditions, fish feeding habits and ecosystem characteristics [32]. The occurrence of blooms and the presence of MCs in water are temporally and spatially variable, leading to different exposure of fish to MCs [33,34]. Inter-species differences in fish are also important factors for the intake and accumulation of MCs in their tissues. For example, zooplanktivorous fish were more likely to accumulate MCs than fish of other feeding guilds e.g., omnivorous, such as the studied *Cyprinous Carpio* [35]. The mechanisms of MC excretion and the timescales for MC elimination can also differ considerably among different fish species [36]. 

In the course of this study, *Microcystis aeruginosa* and *Aphanizomenon ovalisporum* were almost unique cyanobacterial species that were observed in autumn/spring and summer, respectively. These results are in agreement with a previous study reporting *Microcystis aeruginosa* and *Aphanizomenon ovalisporum* to be the most frequently encountered bloom-forming species in Lake Karaoun, either separately or together [14]. Furthermore, low phytoplankton biodiversity in Lake Karaoun was reported by Fadel and Slim who found that, since 2009, these two species had constituted >95% of the total phytoplankton biomass [16]. It was also reported that in Lake Karaoun, *Aphanizomenon ovalisporum* blooms formed both in spring and autumn, while *Microcystis aeruginosa* blooms formed at higher water temperatures observed during the summer months [15]. Although, *Aphanizomenon ovalisporum* is a well-known CYN producer, CYN was not detected during this study. On the contrary, the presence of CYN was previously reported during May to November 2012 and March to May 2013. The determination was performed with ELISA showing concentrations ranging from 0.5 to 1.7 μg/L in 2012, and from undetected to 1.7 μg/L in 2013 [17]. However, to date, the detection of CYN has never been confirmed by LC-MS/MS. 

Monitoring studies of lakes and reservoirs in the Middle East are rare, with the exception of the closest natural lake, Lake Kinneret (Israel). Although Lake Kinneret is less eutrophic than the Karaoun reservoir, blooms of *Microcystis sp.* have been reported since the late 1960s [37], while *Aphanizomenon ovalisporum* and *Cylindrospermopsis raciborskii* were first detected in September 1994 and summer 1998, respectively [38,39]. The most frequently found cyanotoxins in Lake Kinneret have been CYN and MCs [21]. Despite the fact that CYN was not detected in this study, it is proposed that CYN should be included, together with MCs, in future monitoring programs to better evaluate the associated risks, since *Aphanizomenon ovalisporum* blooms continue to occur in lake Karaoun. NOD is mostly associated with brackish water cyanobacteria with a limited number of studies to report its occurrence in freshwater bodies [4,40]. Therefore, its presence is generally not expected in Lake Karaoun, while it can be determined along with MCs due to its structural similarity with them [41] in future monitoring programs. The presence of ATX should further be investigated, in cases where known cyanobacteria producers are present [4]. 

Screening for volatile compounds showed the presence of T&O cyanobacterial/algal terpenoids β-cyclocitral (in 3 samples) and β-ionone (in 3 samples). In particular, the presence of β-cyclocitral was reported to be associated with the occurrence of strains of *Microcystis* [42,43] which has a tobacco-woody odor with a rather high odor threshold concentration of 5 μg/L [44]. It was shown that β-cyclocitral is rapidly produced upon *Microcystis* cell rupture via a carotene oxygenase reaction and it subsequently affects grazer behavior, acting as a repellent and signal of low-quality food to grazers [43]. β-Ionone, commonly occurring in algae and higher plants, is also produced through the carotenoid cleavage of dioxygenases [45]. It has a characteristic flowery-woody odor, showing strong odor thresholds, at a level of 0.007 μg/L [46]. The release of β-ionone in lake water was positively correlated with microcystis biomass [47]. The common cyanobacteria/actimomycetes T&O compounds, geosmin and 2-methylisoborneol were not detected in any of the samples.

Nonanal, a known cyanobacterial/algal aldehyde with fishy odor [24] and an odor threshold of 1 μg/L in water [48] was detected in 9 samples. Aldehydes are mostly derivatives of polyunsaturated fatty acids and are common causes of odor in surface waters [18]. Dimethyl disulfide, dimethyl trisulfide and dimethyl tetrasulfide were detected in sample 4 that had a strong swampy/septic odor. Such undesirable odors have been a major concern for drinking water in several countries. In particular, dialkyl sulfides present strong odors described as swampy/septic, rotten, rancid and stinky, with very low odor threshold concentration levels of ng/L or less [49,50,51]. These and other organosulfur compounds can be produced both under oxic and anoxic conditions by a diversity of biota, biochemical pathways, enzymes and precursors [52]. 3-methylindole (skatole) was detected in samples 2 and 4. In particular, sample 4 had a strong swampy/septic odor due to the presence of dimethyl sulfides and skatole. The latter is a fecal compound with a characteristic odor that was previously reported to occur in algal cultures and field samples [18]. 

Several species of cyanobacteria can produce cyanotoxins and T&O compounds. For example, some strains of *Microcystis* produce microcystins together with β-cyclocitral and alkyl sulfides [42]. However, cyanobacterial T&O do not inevitably indicate the occurrence of cyanotoxins [53]. Nevertheless, since T&O can be sensed by the human nose at very low concentrations, they can serve as an early warning indicator for further investigations into the presence of toxic cyanobacteria [54]. The cyanobacterial/algal T&O detected in this study can serve as an initial list of compounds to be screened in future T&O episodes in Beirut’s drinking water supplies. 

The hydrocarbons detected, such as 2,2,4,6,6-pentamethyl heptane (all samples) and straight-chain hydrocarbons hexadecane (2 samples) and heptadecane (6 samples), could generally be of anthropogenic or biogenic origin. The hydrocarbon 2,2,4,6,6-Pentamethyl heptane has many industrial uses in anti-freeze products, coatings, fillers, lubricants, greases, etc. [55]. On the other hand, cyanobacteria and algae have long been known producers of alkanes, and their potential for biofuel production has been an area of increased research interest [56]. A study of volatile compounds associated with cyanobacteria and algae in freshwater by Juttner et al., [42] reported that biogenic hydrocarbons were represented exclusively by straight-chain components. Other compounds detected, such as 2,4-di-*tert*-butylphenol (antioxidant), diisobutyl phthalate (plasticizer) and p-cresol are common industrial pollutants, with uses in fuels, plastics, and production of chemicals. These were identified in samples 10, 4 and 2, respectively. 

Quantitative determination of VOCs showed the presence of dichloromethane in all samples, at concentrations up to 34 μg/L. Dichloromethane is a common industrial solvent used in many chemical processes. Toluene was detected in the majority of samples at concentrations of up to 0.81 μg/L, while in sample 16 it co-occurred with traces of benzene and o,m,p-xylenes. These compounds are found in fuels and petroleum products and are also common industrial solvents. The “BTEX” volatiles (benzene, toluene, ethylbenzene, xylenes) are frequently used as an index to assess the impact of pollution caused by spills or leaks from fuel storage tanks into water bodies. Trichloromethane, detected in two samples at concentrations of up to 0.5 μg/L could indicate contamination of the lake with wastewater, since trihalomethanes, are commonly present in chlorine-treated water [57]. 

The above findings imply that, besides cyanobacteria and their metabolites, cyanotoxins and T&O compounds, anthropogenic pollution can also be a concern for Lake Karaoun, regarding its use as a drinking water reservoir, supporting the emerging need for studies and impact assessment of the co-occurrence of toxic cyanobacteria with other anthropogenic pollutants [58]. 

## 4. Conclusions

The results of this study demonstrate for the first time the presence of multiple MC congeners in Lake Karaoun, i.e., dmMC-RR, MC-RR, MC-YR, dmMC-LR, MC-LR, MC-HilR, MC-WR, MC-LA, MC-LY, MC-LW, and MC-LF, with total MCs reaching up to 429 μg/L (sample 16). Additionally, T&O compounds such as β-cyclocitral, β-Ionone, nonanal and dimethylsulfides were identified, while industrial pollutants of anthropogenic origin including dichloromethane and toluene were determined up to 34 (sample 9) and 0.81 (sample 16) μg/L, respectively. Complementary methods were applied aiming at the reliable determination of cyanotoxins and T&O compounds in the lake. Since blooms of *Microcystis aeruginosa* and *Aphanizomenon ovalisporum* continue to occur in Lake Karaoun, monitoring of cyanobacterial blooms is necessary in the future, for the assessment of risks related to the intended uses of the lake. With regard to the cyanotoxin analysis, the results show that MCs should be a monitored target, especially when blooms of *Microcystis aeruginosa* occur. Quantitative screening by ELISA or PPIA could be used for the monitoring of MCs if LC-MS/MS facilities and expertise are not available. It is, however, recommended to confirm the findings by LC-MS/MS which has the advantage of being compound-specific. Despite the fact that CYN was not detected in this study, CYN should be included in the ongoing monitoring schemes, especially in the presence of *Aphanizomenon ovalisporum* in the lake. Whenever incidents of unpleasant odors occur in the lake or in water supplies, further analysis of T&O and VOCs should be carried out to identify the source of T&O. 

## 5. Materials and Methods

### 5.1. Study Site and Sampling

**Figure 4:**
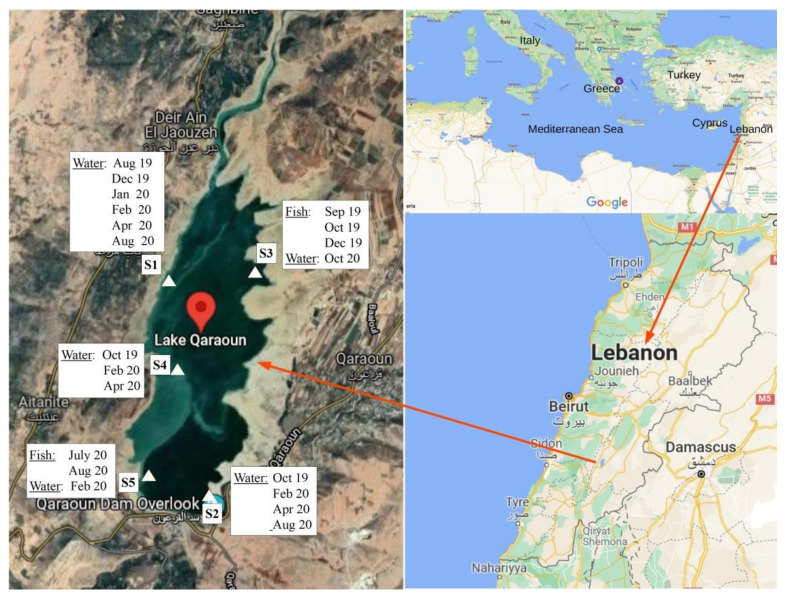
Map and sampling points of Lake Karaoun, Lebanon.

The Middle-Eastern Lake Karaoun (Qaraoun) is the largest water body in Lebanon, located in the western part of the valley Bekaa (33.34° N, 35.41° E) (Figure 4). Lake Karaoun is an artificial reservoir, created in 1959 by construction of a dam on the Litani River. It has a surface of 12 km^2^ at a full capacity of 220 million m^3^, a maximum depth of 60 m and a mean depth of 19 m [16]. The lake is considered to be located in a vulnerable arid to semi-arid zone, with winters being moderately cold (13 °C average temperature), the wet season extending from November to April, and summers being hot and dry, lasting from July to October [15,59].

During field campaigns that covered the wet and dry periods, 16 water samples along with a total of 9 specimens of *Cyprinus carpio* fish (weighing 215 g on average) were collected mainly by the national Litani River Authority. All water samples were collected in polyethylene bottles from 5 sampling points of Lake Karaoun (S1, S2, S3, S4 and S5). S1 was located close to the river input at the west bank, S2 was east of the dam, S3 was close to the river input at the east bank, S4 was in the middle of the lake and S5 was at the dam (Figure 4). Water samples were transported in coolers (at a temperature of 4 °C) for laboratory analysis in Beirut, Lebanon and Athens, Greece. The fish were kept on ice and were sacrificed within 24 h. Fish liver and muscle sub-samples were labeled and frozen separately at −20 °C. The frozen fish tissue samples were then lyophilized with a Labconco freeze-dryer for 48 h at −84 °C and 0.2 mbar and powdered using a pestle and a mortar.

### 5.2. Chemicals and Reagents

Cyanotoxin standards of MC-RR, MC-LR, MC-YR, MC-LA and NOD were purchased from Sigma-Aldrich (Steinheim, Germany), [D-Asp3]MC-LR, [D-Asp3]MC-RR, MC-WR, MC-HtyR, MC-HilR, MC-LY, MC-LW and MC-LF from ENZO Life Science (Lausen, Switzerland), CYN from Abraxis (Warminster, PA, USA), and ATX fumarate from TOCRIS Bioscience (Bristol, UK). All toxin standards had purity >95%. A VOC 57 standard mix (44926-U) was purchased from Supelco (Darmstadt, Germany). β-Cyclocitral (C_10_H_16_O) (≥95.0%), β-ionone (C_13_H_20_O) (purity ≥ 97.0%), pentadecane (C_15_H_32_) (purity ≥ 99.8%), 2,4-di-*tert*-butylphenol (C_14_H_22_O) (99.0%), hexadecane (C_16_H_34_) (purity ≥99.8%), heptadecane (C_17_H_36_) (purity ≥ 99.5%), dimethyl disulfide (CH_3_SSCH_3_) (≥99.0%), dimethyl trisulfide (purity ≥ 98.5%) and 3-methylindole (C_9_H_9_N) (98.0%), were supplied by Sigma Aldrich (Steinheim, Germany). Acetonitrile (ACN) and methanol (MeOH) of HPLC grade (≥99.9%) as well as dichloromethane (DCM) and hexane (HXN) of analytical grade (99.9%) were supplied by Fisher Chemical (Loughborough, UK). High purity formic acid (HCOOH) (98–100%) and acetic acid (CH_3_COOH) (>99.8%) were obtained from Riedel-de Haën (Seelze, Germany). Sodium chloride (NaCl) of analytical purity (99.5%) and fuming (37%) hydrochloric acid (HCl) were purchased from Merck (Darmstadt, Germany). Ethylenediaminetetraacetic acid (EDTA) of analytical grade was supplied from Serva Electrophoresis. Sodium hydroxide (NaOH) 2M solution used for adjustment of pH was prepared from NaOH pellets (purity 98%) purchased from Sigma-Aldrich (Steinheim, Germany). Potassium carbonate (K_2_CO_3_) (>99.5%) was supplied from Carlo Erba Reagents. High purity water (18.2 MΩ cm at 25 ^o^C) was produced in-house using a TEMAK TSDW10 water purification system (TEMAK, Athens, Greece).

### 5.3. Microscopic Examination

Microscopic examination of samples was carried out within 24 h on 20 mL water samples collected during field campaigns with a phytoplankton net and kept at low temperature (4 °C). Examination was carried out in the LAEC laboratory in Beirut, Lebanon using a phase contrast microscope (Olympus, Munster, Germany), under a ×40 objective and ×100 immersion. Identification of cyanobacteria was based on taxonomic keys as cells structures and dimensions, mucilage features and the form of colonies. Estimation of cyanobacterial abundances was performed as described elsewhere [17]. 

### 5.4. LC-MS/MS Analysis of Cyanotoxins

#### 5.4.1. Sample Preparation of Water Samples

Preparation of water samples for LC-MS/MS analysis of cyanotoxins was based on the method of Zervou et al. [22]. For the determination of extracellular toxins, 150 mL of sample was filtered using a glass fiber filter (Millipore, Ireland). After adjusting the pH of the filtrate to 11, solid phase extraction (SPE) was performed using a dual cartridge assembly with a Supel-Select HLB (bed wt. 200 mg, volume 6 mL, Supelco) and a Supelclean ENVI-Carb (bed wt. 250 mg, volume 3 mL, Supelco) on a 12-port SPE vacuum manifold (Supelco) connected with a vacuum pump. Conditioning of cartridges was carried out with 6 mL DCM, followed by 6 mL of MeOH and 6 mL of pure water (pH 11). Sample passed through the two tandem cartridges at a 0.5 mL/min flow rate. After sample passing, cartridges were dried for 15 min (air under vacuum) and the sequence of cartridges in the assembly was reversed. Elution was done with (60:40) MeOH/DCM having 0.1% HCOOH. Eluents were evaporated to dryness under a gentle nitrogen stream, reconstituted with 150 µL of 5% (*v/v*) MeOH, and transferred into vials for LC-MS/MS analysis. For the determination of intracellular cyanotoxins, after sample filtration the filters were extracted, according to Chistophoridis et al. [23], with 9 mL 75% (*v/v*) MeOH. A 3-mL aliquot of the filtered supernatant was evaporated to dryness and the residue was re-dissolved with 500 µL of 5% (*v/v*) MeOH for LC-MS/MS analysis.

#### 5.4.2. Sample Preparation of Fish Samples

Subsamples of 0.2 g of lyophilized powdered flesh or 0.25 g liver were extracted with 5 mL 80% MeOH containing 0.5% HCOOH by stirring for 15 min, followed by ultra-sonication for 30 min (Bandelin Sonorex Super RK106). The mixture was then transferred to a Falcon tube and centrifuged for 15 min at 4500 rpm (DuPont RMC-14 Refrigerated Micro-Centrifuge, Sorvall Instruments, Newtown, CT, USA). The supernatant was extracted three times with 1 mL of hexane. The hexane phase was discarded, and the bottom layer was transferred to a flask and diluted with 100 mL of water containing 0.3% formic acid. The extract was cleaned-up by SPE with a Supel-Select HLB cartridge (bed wt. 200 mg, volume 6 mL, Supelco) conditioned with 6 mL MeOH followed by 6 mL acidified water (0.3% HCOOH). After extraction, the cartridge was washed with 6 mL of water, dried for 15 min under vacuum and eluted with 6 mL MeOH. The eluents were dried in a water bath at 40 °C under a gentle nitrogen stream. Reconstitution was carried out with 200 µL of 5% MeOH, followed by vortexing and sonication for 3 min, prior to LC-MS/MS analysis.

#### 5.4.3. Determination by LC-MS/MS

A Finnigan Surveyor LC system, equipped with an AS autosampler (Thermo, Waltham, MA, USA) coupled with a TSQ Quantum Discovery Max triple-stage quadrupole mass spectrometer (Thermo, Waltham, MA, USA) with electrospray ionization (ESI) source, was used for LC-MS/MS analysis. Data was acquired and processed by Xcalibur software. Targeted analysis of CYN, ATX, NOD and 12 MCs (dmMC-RR, MC-RR, MC-YR, MC-HtyR, dmMC-LR, MC-LR, MC-HilR, MC-WR, MC-LA, MC-LY, MC-LW, MC-LF) was performed as described in a previous study [22]. Detection of CTs was carried out in multiple reaction monitoring (MRM) mode, using the three most intense and characteristic precursor/product ion transitions for each toxin. Confirmation of identity was based on criteria for retention time (t_R_), characteristic precursor/product ion transitions and two calculated ratios of precursor/product ion transitions. LC-MS/MS detection parameters of targeted cyanotoxins are given in Table S3. An example of MC-LR identification in a sample (S2, 2 October 2019) from Lake Karaoun is shown in Figure S4.

### 5.5. ELISA for Microcystins

ELISA was carried out with the Microcystins-ADDA ELISA kit (Eurofins—Abraxis, Warminster, PA, USA) in 96 well microplates, using an Infinite M200 reader (Tecan, Männedorf, Switzerland). The kit was used according to the manufacturer’s instructions and concentrations of toxins were calculated using calibration curves based on the absorbance at 450 nm. For the determination of extracellular MCs, water samples were filtered through 47 mm glass fiber filters (Millipore) and analyzed without any further treatment. Dilutions with ELISA sample diluent were carried out when samples exceeded 5 μg/L MC-LR equivalents. For the analysis of intracellular MCs, after sample filtration (see 5.4.1) the filter was extracted with 9 mL of 75% MeOH. Then, 3 mL of the extract was evaporated to dryness and the residue was re-dissolved in water to avoid false results due to the high percentage of MeOH [41]. Finally, samples were analyzed in duplicate and mean values were reported when RSD < 25%.

### 5.6. Protein Phosphatase Inhibition Assay (PPIA) for Microcystins

PPIA was carried out using the Microcystins/Nodularins PP2A kit (Eurofins—Abraxis) in 96 well microplate using an Infinite M200 reader (Tecan, Männedorf, Switzerland) for measurements at 405 nm, according to manufacturer’s instructions. Dilutions with ultrapure water were carried out when samples exceeded 2.5 μg/L MC-LR equivalents. Sample preparation prior to PPIA was the same as in ELISA. Samples were analyzed in duplicate and mean values were reported when RSD < 25%.

### 5.7. qPCR Assay for Total Cyanobacteria and Cyanotoxin Genes

The Phytoxigene™ CyanoDTec (Diagnostic Technology, Belrose, Australia) multiplex quantitative real-time PCR assay was applied to determine the gene copies of the 16s rRNA gene (total cyanobacteria) and the *mcyE*, *cyrA*, *sxtA* genes (microcystins, cylindrospermopsin, saxitoxins, respectively). The kit was used according to the manufacturer’s instructions and PCR was carried out in a Smartcycler II system (Cepheid). In brief, a volume of water sample (1 to 15 mL, depending on visual cell density) was filtered through a Nucleopore 25 mm, 0.8 µm filter (Whatman, Little Chalfont, UK) using a syringe and filter holder. DNA extraction of filters was carried out using BioGx bead lysis tubes (Diagnostic Technology) in a BeadBug bead beater (Benchmark Scientific). Extracts were centrifuged (MicroCL 21, Thermo Fisher Scientific, Waltham, MA, USA) and proceeded to qPCR. Quantitation of gene copies was based on calibrations with the Phytoxigene™ CyanoNAS standards (100–1,000,000 copies per reaction). 

### 5.8. GC-MS Analysis of Volatile Compounds

Analysis of volatile compounds was carried out in order to (a) screen samples to detect and identify typical cyanobacterial volatile and T&O compounds (untargeted method) and (b) to quantitatively determine a range of anthropogenic VOC pollutants (targeted method). 

#### 5.8.1. Untargeted Screening of Cyanobacterial Volatiles and T&O Compounds

Headspace Solid Phase Microextraction coupled to Gas Chromatography-Mass Spectrometry (HS-SPME-GC-MS) was used to screen water samples for volatile compounds with focus on cyanobacterial T&O. A 456 GC coupled to TQ mass spectrometer and equipped with an SPME autosampler (Bruker Daltonics, Bremen, Germany) was used. A (2–10 mL) aliquot of the sample was transferred into a 20 mL SPME glass vial containing 3 g NaCl, then adjusted to 10mL with ultrapure water and tightly sealed. HS-SPME was carried out automatically under the following conditions: 2 cm Divinylbenzene/Carboxen/Polydimethylsiloxane SPME fiber (Supelco, Bellefonte, PA, USA), equilibration 10 min at 60 ^o^C, headspace extraction: 10 min at 60 ^o^C, agitation 300 rpm and desorption time 2 min. GC analysis was carried out using a column RXI^®^- 5 Sil MS, 30 m, 0.25 mm ID, 0.25 µm df (Restek, Bellefonte, PA, USA). GC conditions were injector temp. 250 °C, splitless, constant flow 1 mL/min (He), column program: (a) 50 °C (1 min) to 250 °C at a rate of 15 °C/min, 250 °C (5 min) and (b) 35 °C (5 min) to 250 °C at a rate of 8 °C/min, 250 °C (5 min). MS conditions were: EI source (70 eV), scan 30–300 m/z, positive polarity. Fluorobenzene standard solution in methanol (Sigma – Aldrich, St. Louis, MO, USA) spiked at a concentration of 5 μg/L in the samples was used as a surrogate for evaluation of SPME efficiency. 

Mass spectrometry data were processed using MSWS software (Bruker). Mass spectral deconvolution and identification of compounds was carried out with AMDIS (NIST, Gaithersburg, MD, USA) using the NIST MS library (2015) and retention index calibration with C7-C30 saturated alkanes reference standard (Sigma). Combined matching scores (retention index-RI and mass spectral matching) were used as criteria for identification (RI ± 20 of the reference RI and a spectral matching ≥80%). Identification was considered definite only if a reference standard of the suspect compound was available in the lab and retention times of the suspect and reference compounds matched within ±0.01 min, in addition to mass spectral matching. 

#### 5.8.2. Quantitative Determination of VOCs

Quantitative determination of a number of VOCs was carried out by HS-SPME-GC-MS according to EN ISO 17943:2016 (HS-SPME-GC-MS), using 5 ml samples [60]. The compounds determined were chloroform, bromoform, dibromochloromethane, bromodichloromethane, benzene, 1,2-dichloroethane, tetrachoroethene and trichloroethene, 2-ethoxy-2-methyl propane (ETBE), bromochloromethane, dibromomethane, toluene, 1,2-dibromoethane, chlorobenzene, styrene, bromobenzene, n-butyl benzene, hexachlorobutadiene, naphthalene, 1,1-dichloroethane, 1,1-dichloroethene, 1,1,2-trichloroethane, 1,1,2,2-tetrachloroethane, 1,2-dibromo-3-chloropropane, 1,2-dichloropropane, 1,2,3-trichlorobenzene, 1,2,4-trichlorobenzene, 1,3-dichloropropane, 2-methoxy-2-methyl-butane (TAME), ethylbenzene, n-propylbenzene, o-xylene, p+m-xylenes, sec-butylbenzene and tert-butylbenzene. A VOC 57 standard mix (Supelco) was used for calibration and fluorobenzene, toluene-d_8_ and benzene-d_6_ (Sigma) as internal standards. HS-SPME conditions were: 2 cm Divinylbenzene/Carboxen/Polydimethylsiloxane SPME fiber (Supelco), equilibration for 10 min at 40 °C, headspace extraction for 10 min at 40 °C, agitation 300 rpm and desorption time 2 min. GC conditions were: column RXI^®^- 624 Sil MS, 60 m, 0.32 mm ID, 1.8 µm df (Restek), injector temp. 250 °C, splitless, constant flow 1ml/min (He), column program: 35 °C (5 min) to 250 °C at a rate of 8 °C/min and at 250 °C (10min). MS conditions were: EI source (70 eV), Selected Ion Monitoring (3 ions per compound), positive polarity. LOD of each compound was 0.2 μg/L. Confirmation of determinations was carried out with the ISO criteria for retention time and ion ratios [60]. 

### 5.9. Method Validation and Quality Control Procedures

The methods applied in this study were validated either previously or in the frame of the study and method performance parameters such as specificity, linearity, precision, accuracy, and limits of detection have been assessed. Furthermore, quality control procedures including measurements of blank/negative and control/positive samples were followed in each batch of analyzed samples. 

Validation data of the method applied for the determination of CTs in water by LC-MS/MS are reported in previous studies with RSD values of detected CTs being <16% and <26% for extra- and intra-cellular fractions, respectively [22,23]. This method is also accredited by ISO 17025 in the NCSR Demokritos laboratory [61]. 

The LC-MS/MS method for determination of CTs in fish was developed, optimized and validated in the frame of this study. For optimization, various combinations of extraction solvents, extraction/mixing times, clean-up steps and SPE cartridges were tested (Oasis HLB and HLB followed by Sep-Pak Vac, silica) as presented in Figures S5 and S6. Optimization experiments were performed with samples spiked with MC-LR and MC-RR at concentration levels of 100 and 400 ng/g dw for flesh and liver, respectively. Selection of the optimized conditions was based on maximization of % recoveries. As shown in Figure S7, method 2 provided the highest mean recoveries for the two spiked MCs in flesh (78.9% for MC-RR and 79.1% for MC-LR) and liver (77.1% for MC-RR and 75.4% for MC-LR). Subsequently, the optimized method was validated in-house. Recovery and precision were evaluated by analyzing toxins-free lyophilized flesh and liver fish samples spiked with a mixture of 12 MCs, at concentration levels of 100 and 400 ng/g dw in 3 replicates. Samples were extracted and analyzed as outlined in Section 5.4.2. Table S4 provides performance characteristics of this method. Briefly, mean recoveries of [D-Asp^3^]MC-RR, MC-RR, MC-YR, [D-Asp^3^]MC-LR, MC-LR, MC-HilR, MC-LA and MC-LY ranged from 68.5% to 81.6% for flesh, while liver recoveries ranged from 61.5% to 72.2%, with intra-day precision in the range of 6.8–16.5% for flesh and 5.5–15.2% for liver. LODs for fish flesh and liver samples ranged from 1.0 to 7.0 ng/g dry weight and from 0.8 to 5.6 ng/g dry weight, respectively.

In-house validation and method performance data of ELISA and PPIA for MCs in water were reported previously [41] and methods have been proven suitable for quantitative screening of MCs. Negative and positive control samples were included in each analysis batch.

The qPCR assay for total cyanobacteria and cyanotoxin genes included evaluation of negative and positive control samples in each batch of samples. The Phytoxigene™ CyanoNAS standards used for calibration and accurate quantitation were commissioned and developed by the National Measurement Institute (NMI), of the Australian Department of Industry which participates in the International Bureau of Weights and Measures (BIPM) Consultative Committee for Amount of Substance (CCQM).

The untargeted HS-SPME-GC-MS screening method for detection and identification of cyanobacterial volatile compounds has been tested with a wide range of compounds that are available in the lab of EYDAP SA and has been shown to be capable of detection/identification at concentrations generally <1μg/L. The method has also been tested successfully in interlaboratory trials for unknown odorous compounds in water. The targeted HS-SPME-GC-MS method for VOCs in water has been fully validated in-house, is accredited by ISO 17025 and has successfully been evaluated in interlaboratory tests. 

## Figures and Tables

**Table 1:** Concentrations (μg/L) of extracellular/intracellular cyanotoxins (CYN, ATX, NOD, 12 MC variants) in water samples collected from Lake Karaoun during 2019–2020, analyzed by LC-MS/MS.

Sample ID	Sample Details	CYN	ATX	dm MC-RR	MC-RR	NOD	MC-YR	MC- HtyR	dm MC-LR	MC-LR	MC-HilR	MC-WR	MC-LA	MC-LY	MC-LW	MC-LF
1	S1, 22/08/19	ND ^1^/NA ^2^	ND/NA	ND/NA	ND/NA	ND/NA	ND/NA	ND/NA	ND/NA	ND/NA	ND/NA	ND/NA	ND/NA	ND/NA	ND/NA	ND/NA
2	S2, 02/10/19	ND/ND	ND/ND	ND/1.60	0.339/26.4	ND/ND	ND/1.67	ND/ND	ND/1.87	0.438/91.5	ND/1.29	<LOQ ^3^/ND	ND/ND	<LOQ/3.29	0.014/10.5	<LOQ/7.48
3	S4, 02/10/19	ND/ND	ND/ND	ND/ND	ND/ND	ND/ND	ND/ND	ND/ND	ND/ND	ND/ND	ND/ND	ND/ND	ND/ND	ND/ND	ND/ND	ND/ND
4	S1, 22/12/19	ND/ND	ND/ND	ND/0.53	0.050/7.42	ND/ND	0.187/8.28	ND/ND	0.050/0.70	0.158/8.43	ND/ND	ND/ND	ND/ND	ND/ND	ND/ND	ND/ND
5	S1, 15/01/20	ND/ND	ND/ND	ND/ND	0.007/ND	ND/ND	ND/ND	ND/ND	ND/ND	ND/ND	ND/ND	ND/ND	ND/ND	ND/ND	ND/ND	ND/ND
6	S4, 04/02/20	ND/ND	ND/ND	ND/ND	ND/ND	ND/ND	ND/ND	ND/ND	ND/ND	ND/ND	ND/ND	ND/ND	ND/ND	ND/ND	ND/ND	ND/ND
7	S5, 15/02/20	ND/ND	ND/ND	ND/ND	ND/ND	ND/ND	ND/ND	ND/ND	ND/ND	ND/ND	ND/ND	ND/ND	ND/ND	ND/ND	ND/ND	ND/ND
8	S5, 17/02/20	ND/ND	ND/ND	ND/ND	0.005/ND	ND/ND	ND/ND	ND/ND	ND/ND	ND/ND	ND/ND	ND/ND	ND/ND	ND/ND	ND/ND	ND/ND
9	S1, 22/02/20	ND/ND	ND/ND	ND/ND	0.007/ND	ND/ND	ND/ND	ND/ND	ND/ND	ND/ND	ND/ND	ND/ND	ND/ND	ND/ND	ND/ND	ND/ND
10	S2, 22/02/20	ND/ND	ND/ND	ND/ND	0.008/ND	ND/ND	ND/ND	ND/ND	ND/ND	ND/ND	ND/ND	ND/ND	ND/ND	ND/ND	ND/ND	ND/ND
11	S1, 15/04/20	ND/ND	ND/ND	ND/ND	ND/ND	ND/ND	ND/ND	ND/ND	ND/ND	ND/ND	ND/ND	ND/ND	ND/ND	ND/ND	ND/ND	ND/ND
12	S2, 22/04/20	ND/NA	ND/NA	ND/NA	0.090/NA	ND/NA	ND/NA	ND/NA	ND/NA	ND/NA	ND/NA	ND/NA	ND/NA	ND/NA	ND/NA	ND/NA
13	S4, 22/04/20	ND/ND	ND/ND	ND/ND	ND/ND	ND/ND	ND/ND	ND/ND	ND/ND	ND/ND	ND/ND	ND/ND	ND/ND	ND/ND	ND/ND	ND/ND
14	S1, 14/08/20	ND/ND	ND/ND	ND/ND	ND/ND	ND/ND	ND/ND	ND/ND	ND/ND	ND/ND	ND/ND	ND/ND	ND/ND	ND/ND	ND/ND	ND/ND
15	S2, 14/08/20	ND/ND	ND/ND	ND/ND	ND/ND	ND/ND	ND/ND	ND/ND	ND/ND	ND/ND	ND/ND	ND/ND	ND/ND	ND/ND	ND/ND	ND/ND
16	S3, 09/10/20	ND/ND	ND/ND	0.069/ND	7.0/0.086	ND/ND	197/1.94	ND/ND	6.9/<LOQ	209/2.18	1.6/ND	ND/ND	3.0/ND	<LOQ/ND	ND/ND	0.021/ND

^1^ ND: Not detected; ^2^ NA: Not analyzed; ^3^ <LOQ: Values higher than limit of detection (LOD) and lower than limit of quantitation (LOQ); LODs and LOQs are given in Table S1.

**Table 2:** Total Extracellular/total intracellular MCs concentrations in water samples from Lake Karaoun by LC-MS/MS (sum of 12 extracellular MCs/sum of 12 intracellular MCs, μg/L), ELISA (μg/L MC-LR equivalents) and PPIA (μg/L MC-LR equivalents).

ID	SAMPLE DETAILS	Total Extracellular/Intracellular MCs		
		LC-MS/MS * sum of MCs, μg/L	ELISA ** μg/L eq.MC-LR	PPIA ** μg/L eq.MC-LR
1	S1, 22/08/2019	ND/NA	ND/NA	ND/NA
2	S2, 02/10/2019	0.83/146	NA/97.2	NA/111
3	S4, 02/10/2019	ND/ND	ND/ND	ND/ND
4	S1, 22/12/2019	0.45/25.4	0.13/30.6	0.25/16.2
5	S1, 15/01/2020	0.007/ND	ND/ND	ND/ND
6	S4, 04/02/2020	ND/ND	ND/ND	ND/ND
7	S5, 15/02/2020	ND/ND	ND/ND	ND/ND
8	S5, 17/02/2020	0.005/ND	0.12/ND	ND/ND
9	S1, 22/02/2020	0.007/ND	ND/ND	ND/ND
10	S2, 22/02/2020	0.008/ND	ND/ND	ND/ND
11	S1, 15/04/2020	ND/ND	0.25/ND	0.39/ND
12	S2, 22/04/2020	0.090/NA	1.80/NA	0.87/NA
13	S4, 22/04/2020	ND/ND	2.30/ND	2.32/ND
14	S1, 14/08/2020	ND/ND	ND/ND	ND/ND
15	S2, 14/08/2020	ND/ND	ND/ND	ND/ND
16	S3, 9/10/2020	425/4.29	190/2.41	290/2.34

ND = Not detected, NA = Not Available. LODs: LC-MS/MS 0.001 μg/L; ELISA 0.10 μg/L; PPIA 0.25 μg/L. * analysis with validated methods [22,23], extracellular toxins RSD < 16%, intracellular toxins RSD < 26%; ** duplicate analysis, RSD < 25%.

**Table 3:** Detection of cyanobacteria genes (16S rRNA) and biosynthetic genes of MCs and NOD (*mcyE),* CYN (*cyrA*) and STX (*stxA*) in water samples from Lake Karaoun (gene copies/mL).

ID	SAMPLE DETAILS	Gene Copies/mL			
		16S rRNA (Cyanobacteria)	*mcyE* (MCs & NODs)	*cyrA* (CYN)	*stxA* (STX)
1	S1, 22/08/2019	NA	NA	NA	NA
2	S2, 02/10/2019	6951375	69857	ND	ND
3	S4, 02/10/2019	NA	NA	NA	NA
4	S1, 22/12/2019	9765354	127870	ND	ND
5	S1, 15/01/2020	NA	NA	NA	NA
6	S4, 04/02/2020	194446	ND	ND	ND
7	S5, 15/02/2020	9808	ND	ND	ND
8	S5, 17/02/2020	54262	ND	ND	ND
9	S1, 22/02/2020	62344	ND	ND	ND
10	S2, 22/02/2020	5093	ND	ND	ND
11	S1, 15/04/2020	40877	ND	ND	ND
12	S2, 22/04/2020	186812	ND	ND	ND
13	S4, 22/04/2020	15466	ND	ND	ND
14	S1, 14/08/2020	112948	ND	ND	ND
15	S2, 14/08/2020	34032	ND	ND	ND
16	S3, 09/10/2020	171308	6921	ND	ND

NA: not available, ND: not detected.

**Table 4:** Compounds detected by untargeted HS-SPME-GC/MS screening, identification level of analysis, chemical group and retention time (t_R_) of detected compounds, number of samples in which they have been identified and their % peak areas.

No	Compound	Identification Level	Chemical Group	t_R_ (min)	Number of Samples	% Peak Area
1	α-Thujene	A	Terpenes/Terpenoids	4.82	2	0.05–0.08
2	2,2,4,6,6-pentamethyl heptane	B	Hydrocarbons	5.43	16	0.03- 0.80
3	2,2,6-trimethyl- cyclohexanone	A	Aldehydes/Ketones	5.95	1	0.004
4	p-Cresol	B	Phenols	6.28	2	0.04–5.55
5	2,4-Dimethyldecane	A	Hydrocarbons	6.59	1	0.17
6	Nonanal	A	Aldehydes/Ketones	6.64	9	0.06–0.43
7	4,7-Dimethylundecane	A	Hydrocarbons	7.62	1	0.01
8	β-Cyclocitral	B,C	Terpenes/Terpenoids	7.90	3	0.05–0.16
9	2,6,6-Trimethyl-1- Cyclohexene- 1-acetaldehyde	A	Terpenes/Terpenoids	8.25	1	0.007
10	6,10-Dimethyl-5,9- Undecadien-2-one	B	Aldehydes/Ketones	9.92	1	0.35
11	β-Ionone	B,C	Terpenes/Terpenoids	10.25	3	0.04–0.38
12	Pentadecane	B,C	Hydrocarbons	10.35	1	0.14
13	2,4-di-tert-butylphenol	A,C	Phenols	10.43	10	0.01–39
14	Hexadecane	B,C	Hydrocarbons	11.16	2	0.20–0.60
15	Heptadecane	B,C	Hydrocarbons	11.94	6	0.07–33
16	Diisobutyl phthalate	B	Phthalates	13.11	4	0.003–1.5
17	Dimethyl disulfide	C,D	Alkyl sulfides	5.81	1	0.02
18	Dimethyl trisulfide	C,D	Alkyl sulfides	12.35	1	0.64
19	Dimethyl tetrasulfide	D	Alkyl sulfides	17.68	1	0.78
20	3-methylindole	C,D	Indoles	20.21	2	0.005–0.02

A: Retention Index (RI) ± 20, spectral matching ≥80%; B: RI ± 20, spectral matching ≥90%; C: matching with a reference standard; D: spectral matching ≥80%.

**Table 5:** Concentration (μg/L) of VOCs detected in at least one sample by targeted HS-SPME-GC/MS analysis.

		Concentration (μg/L)									
Sample ID	Sample Details	1,2,3-Trichlorobenzene	1,2,4-Trichlorobenzene	Dichloromethane	Toluene	1,2-Dichloropropane	Benzene	Trichloromethane	o-Xylene	m+p-Xylene	Styrene
1	S1, 22/08/19	ND	ND	31.0	0.72	ND	ND	ND	ND	ND	ND
2	S2, 02/10/19	ND	ND	28.8	0.65	ND	ND	ND	ND	ND	ND
3	S4, 02/10/19	ND	ND	28.1	0.60	ND	ND	ND	ND	ND	ND
4	S1, 22/12/19	ND	ND	29.1	0.25	ND	ND	ND	ND	ND	ND
5	S1, 15/01/20	ND	ND	29.0	<0.25	ND	ND	ND	ND	ND	ND
6	S4, 04/02/20	<0.25	<0.25	27.2	<0.25	ND	ND	ND	ND	ND	ND
7	S5, 15/02/20	ND	ND	28.2	ND	ND	ND	ND	ND	ND	ND
8	S5, 17/02/20	ND	ND	26.3	<0.25	ND	ND	ND	ND	ND	ND
9	S1, 22/02/20	ND	ND	34.0	<0.25	ND	ND	ND	ND	ND	ND
10	S2, 22/02/20	ND	ND	29.4	<0.25	ND	ND	ND	ND	ND	ND
11	S1, 15/04/20	ND	ND	27.2	ND	ND	ND	0.40	ND	ND	ND
13	S4, 22/04/20	ND	ND	30.2	ND	ND	ND	ND	ND	ND	ND
14	S1, 14/08/20	ND	ND	32.3	0.46	ND	ND	ND	ND	ND	ND
15	S2, 14/08/20	ND	ND	33.6	0.35	ND	ND	ND	ND	ND	ND
16	S3, 09/10/20	ND	ND	25.1	0.81	<0.25	<0.25	0.50	<0.25	<0.25	<0.25

ND: Not Detected. Sample 12 was not available for analysis.

## Data Availability

Data is contained within the article or Supplementary Material.

## Supplementary Materials

The following are available online at https://www.mdpi.com/article/10.3390/toxins13100716/s1, Figure S1. chemical structures of studied cyanotoxins, Figure S2. LC-MS/MS MRM chromatogram of sample S2, 02/10/2019 (intracellular fraction) from Lake Karaoun, Figure S3. MRM chromatogram of (a) a standard solution of 12 MCs at a concentration level corresponding to 50 ng/g dw and (b) liver sample from *Cyprinous Carpio* fish collected in September 2019, Figure S4. Example of identification of MC-LR in sample S2, 2 October 2019 (intracellular fraction) from Lake Karaoun, Figure S5. Experimental procedures tested in order to optimize the extraction of MCs in fish flesh, Figure S6. Experimental procedures tested in order to optimize the extraction of MCs in fish liver, Figure S7. Selection of method for (a) fish flesh and (b) fish liver: obtained recoveries of spiked MCs using different treatment processes; Table S1. Method LODs and LOQs for each cyanotoxin analysed in this study using LC-MS/MS, Table S2. Results of untargeted HS-SPME-GC/MS screening per sample, Table S3. LC-MS/MS detection parameters of targeted cyanotoxins, Table S4. Performance characteristics of the method for the analysis of target MCs in fish flesh and liver. Table S5. Physicochemical parameters of Lake Karaoun.
