# Supplementary material for: Investigation of the Occurrence of Cyanotoxins in Lake Karaoun (Lebanon) by Mass Spectrometry, Bioassays and Molecular Methods
Source: Toxins (Basel). 2021 Oct 10;13(10):716. doi: 10.3390/toxins13100716 (PMC8540339; doi:10.3390/toxins13100716)
Supplement: Supplementary file 1 [file toxins-13-00716-s001.zip › Supplementary material_final updated.pdf]

Supplementary Materials: Investigation of the Occurrence of Cyanotoxins in Lake Karaoun (Lebanon) by Mass Spectrometry, Bioassays and Molecular Methods

Noura Alice Hammoud, Sevasti-Kiriaki Zervou, Triantafyllos Kaloudis, Christophoros Christophoridis, Aikaterina Paraskevopoulou, Theodoros M. Triantis, Kamal Slim, Joanna Szpunar, Ali Fadel, Ryszard Lobinski and Anastasia Hiskia

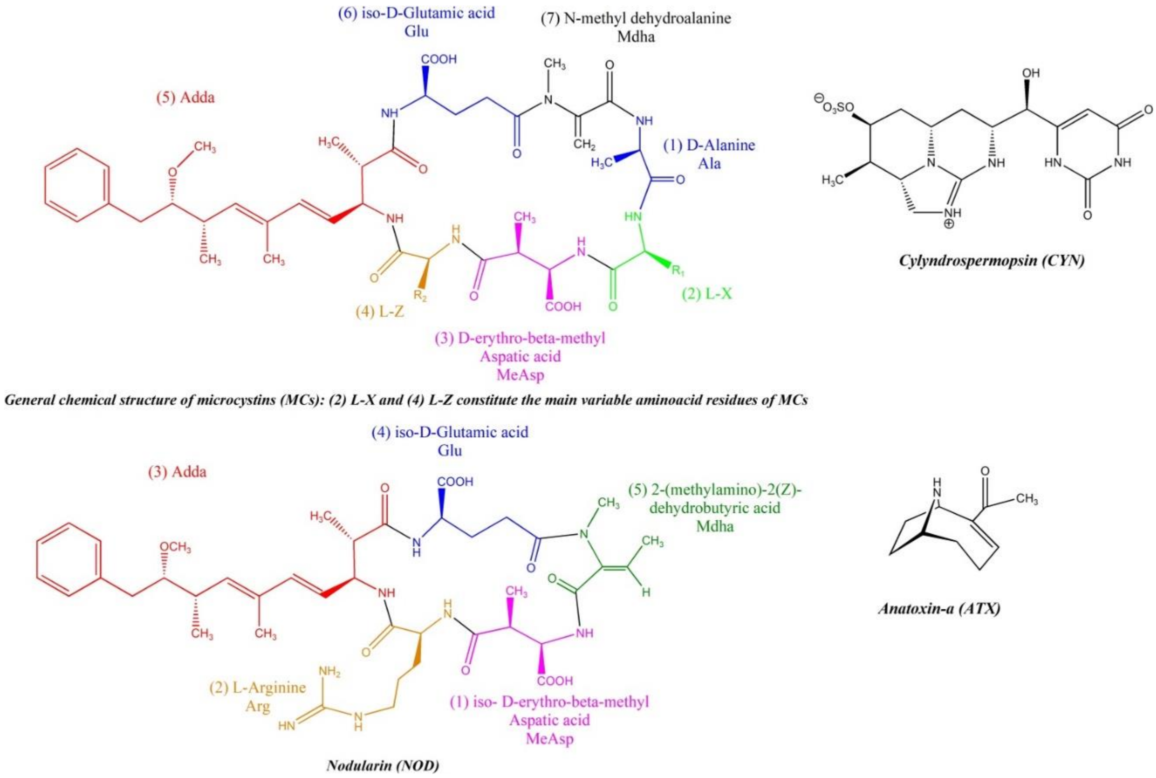

Figure S1. Chemical structures of studied cyanotoxins.

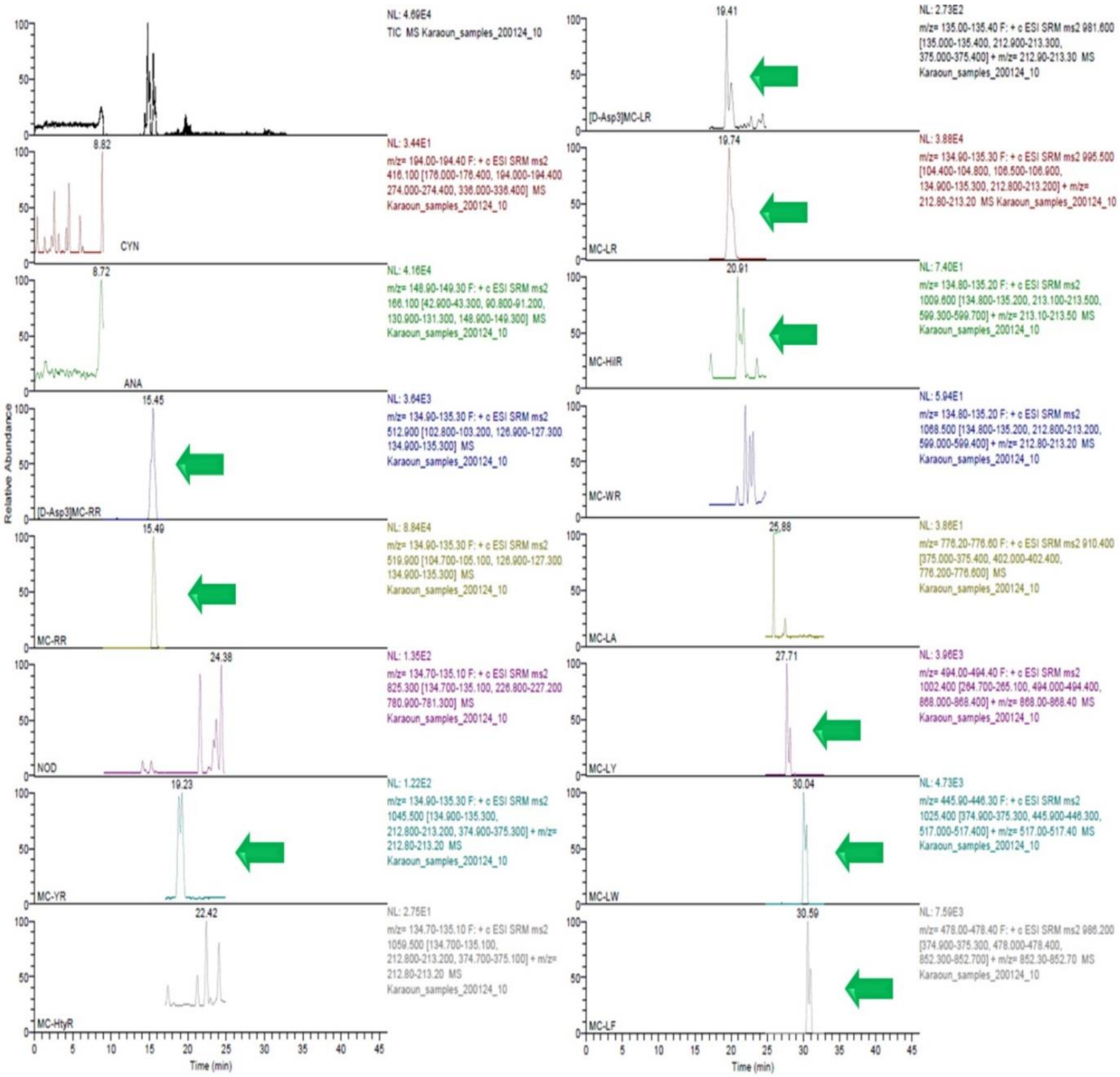

Figure S2. LC-MS/MS MRM chromatogram of sample S2, 02/10/2019 (intracellular fraction) from lake Karaoun.

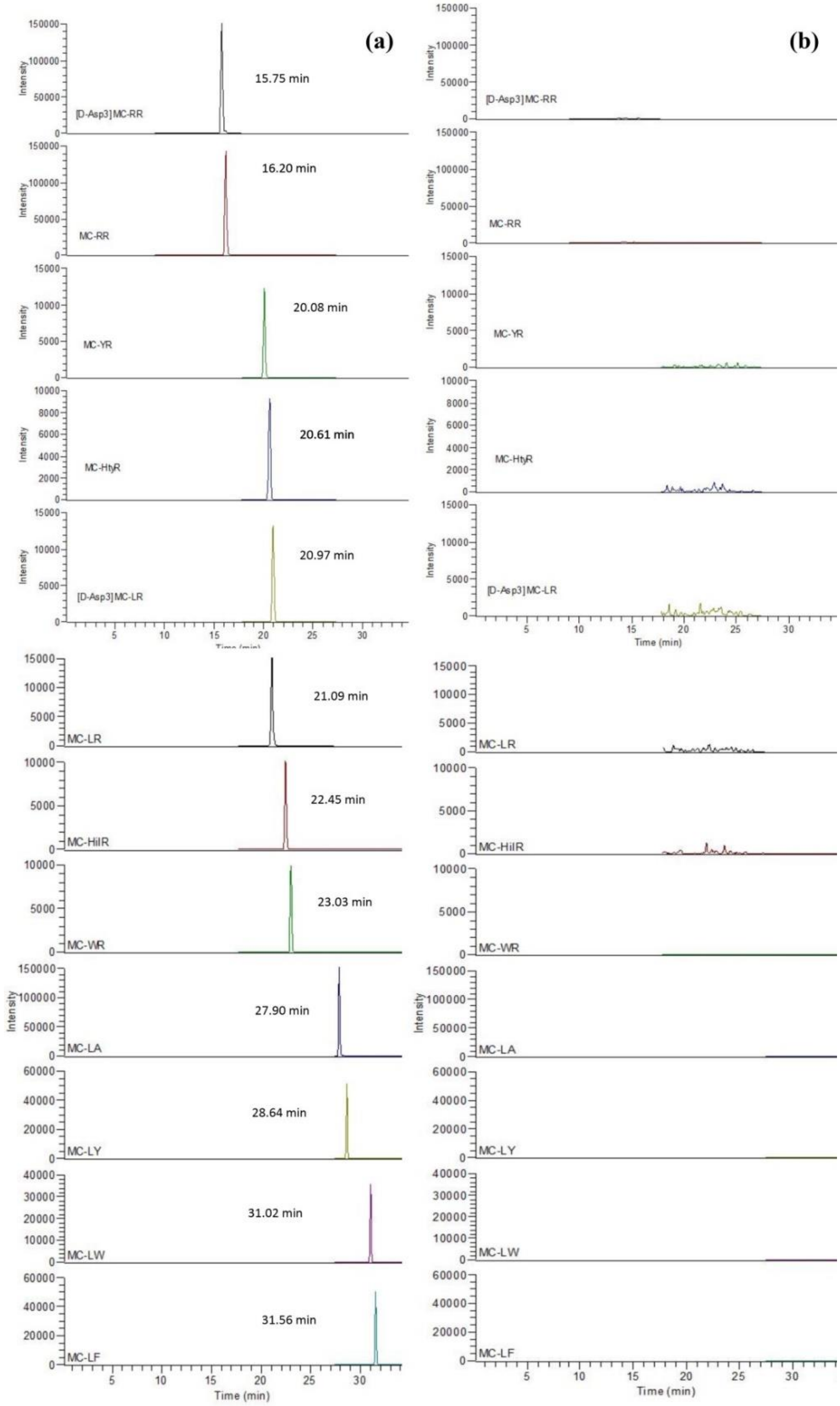

Figure S3. MRM chromatogram of (**a**) a standard solution of 12 MCs at a concentration level corresponding to 50 ng/g dw and (**b**) liver sample from *Cyprinous Carpio* fish collected in September 2019.

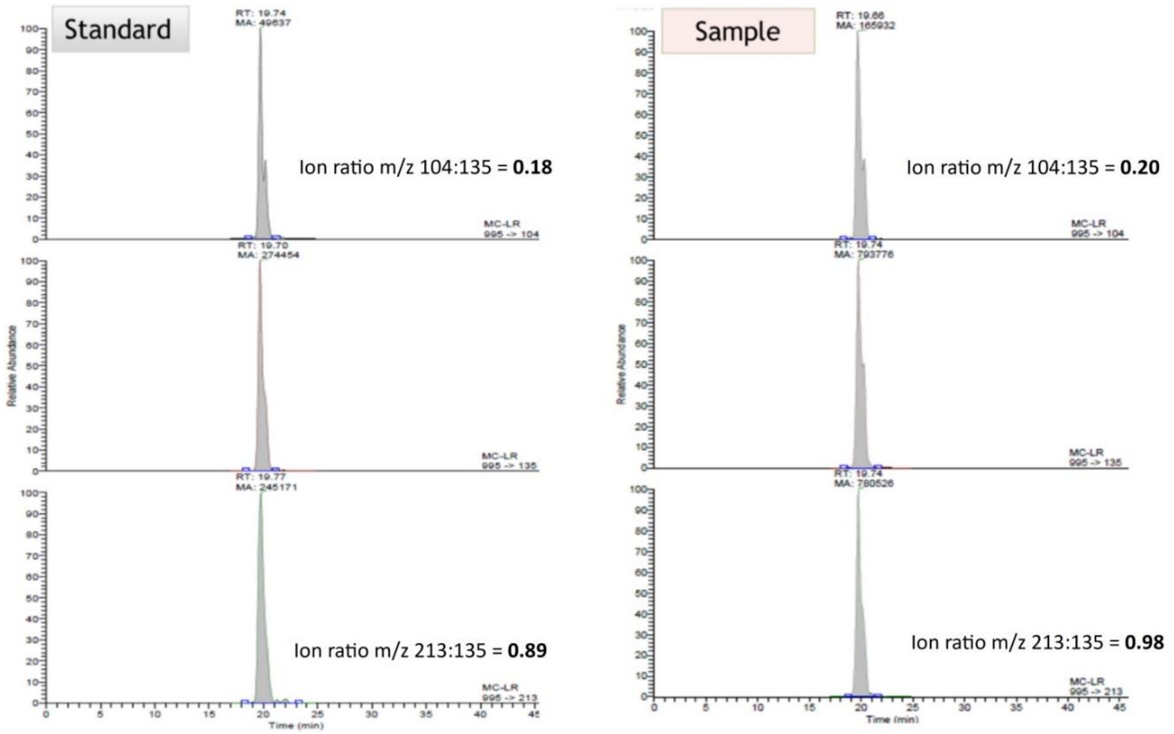

Figure S4. Example of identification of MC-LR in sample S2, 02/10/2019 (intracellular fraction) from lake Karaoun.

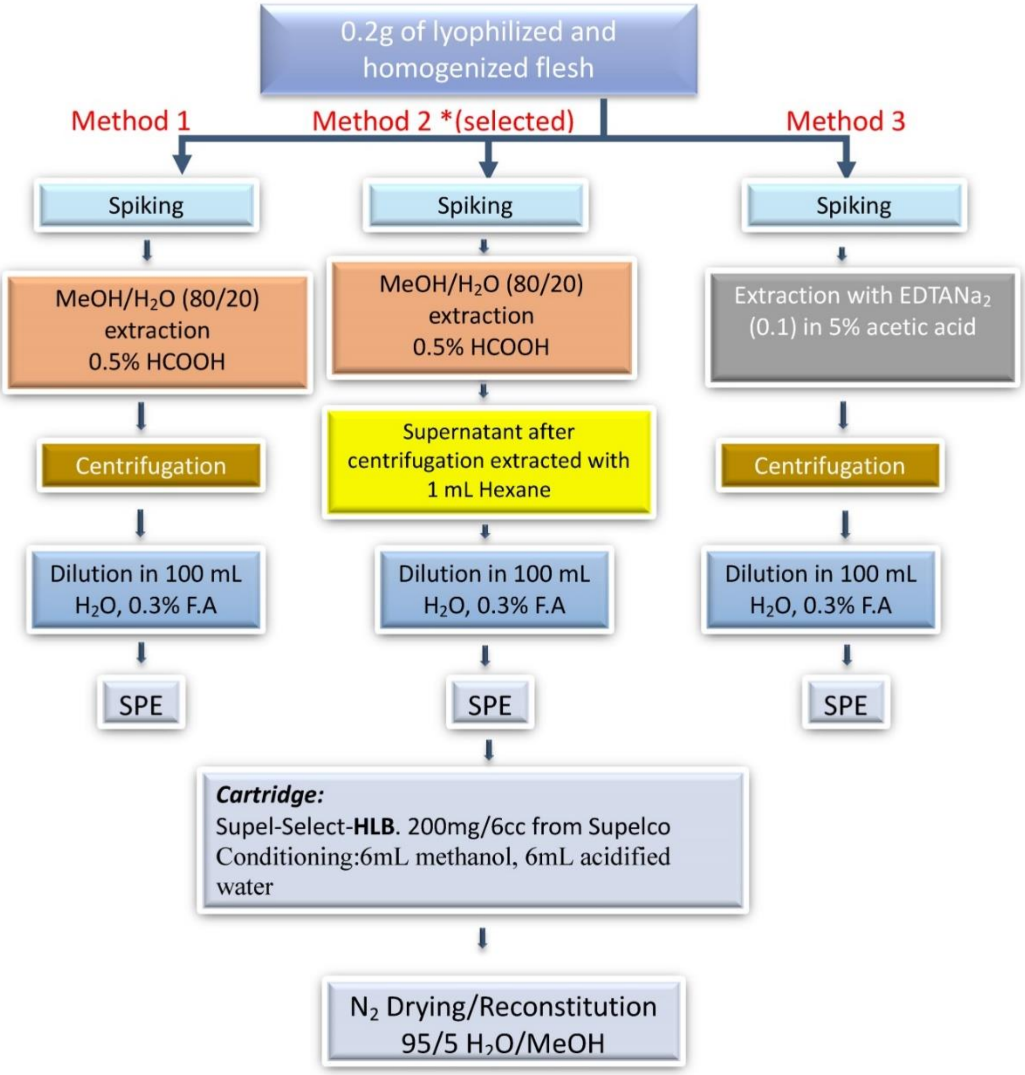

Figure S5. Experimental procedures tested in order to optimize the extraction of MCs in fish flesh.

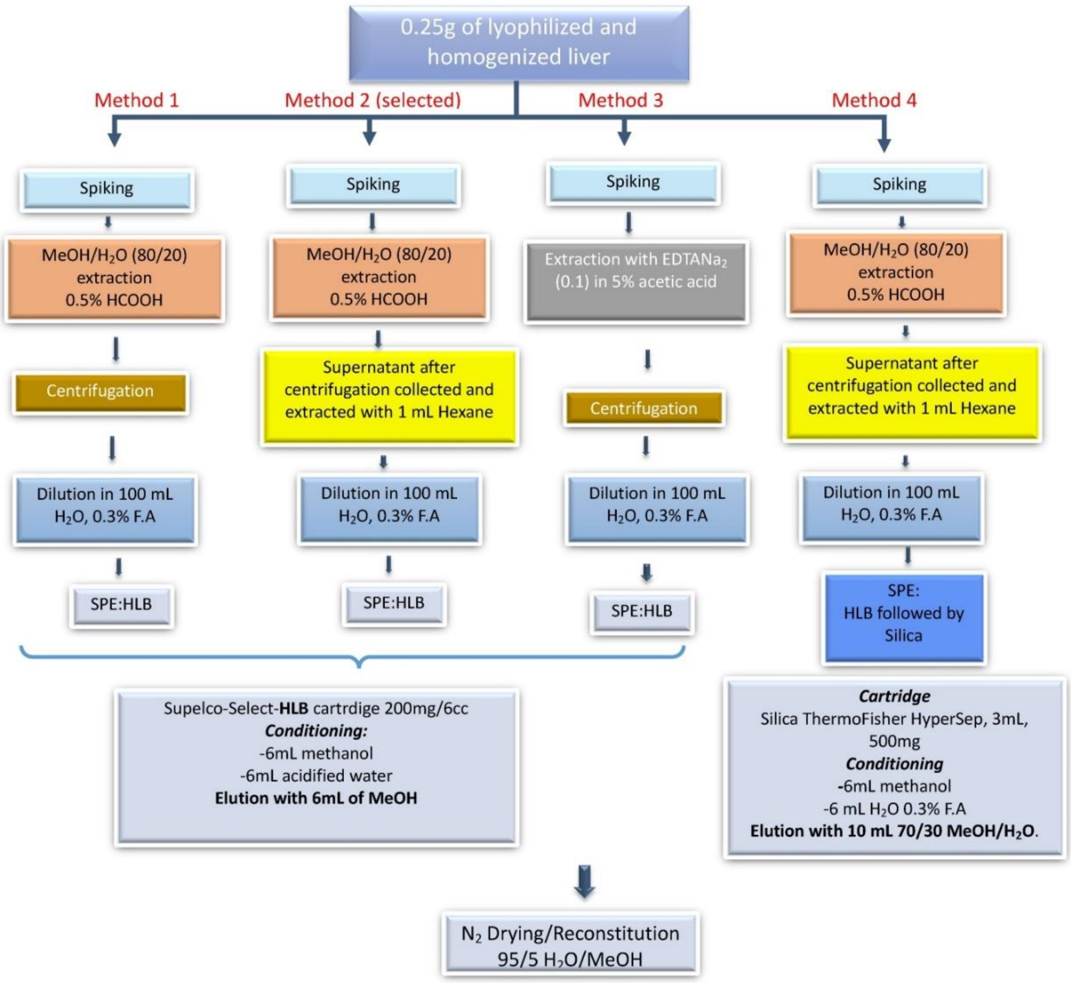

Figure S6. Experimental procedures tested in order to optimize the extraction of MCs in fish liver.

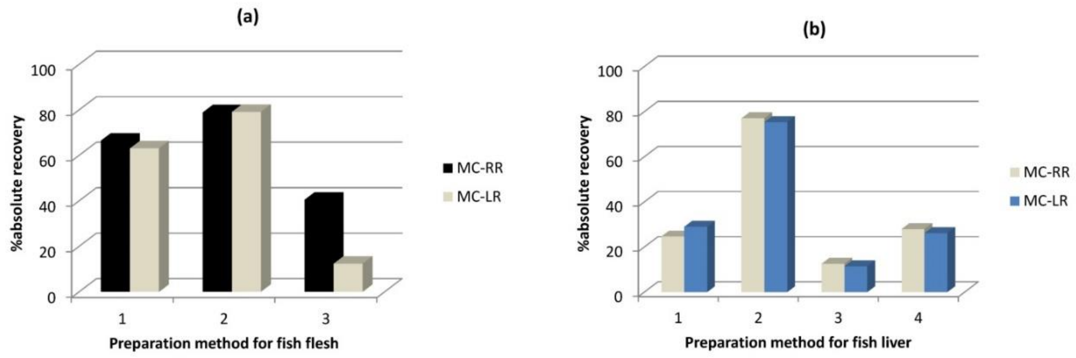

Figure S7. Selection of method for (**a**) fish flesh and (**b**) fish liver: obtained recoveries of spiked MCs using different treatment processes.

Table S1. Method LODs and LOQs for each cyanotoxin analysed in this study using LC-MS/MS.

**Cyanotoxin**	**Extracellular (μg/L)**		**Intracellular (μg/L)***	
	**LOD**	**LOQ**	**LOD**	**LOQ**
CYN	0.001	0.003	0.010	0.030
ATX	0.001	0.003	0.010	0.030
[D-Asp^3]MC-RR	0.002	0.006	0.020	0.060
MC-RR	0.001	0.003	0.010	0.030
NOD	0.002	0.006	0.020	0.060
MC-YR	0.004	0.013	0.040	0.120
MC-HtyR	0.007	0.020	0.070	0.210
[D-Asp^3]MC-LR	0.004	0.011	0.040	0.120
MC-LR	0.004	0.013	0.040	0.120
MC-HilR	0.006	0.019	0.060	0.180
MC-WR	0.006	0.017	0.060	0.180
MC-LA	0.003	0.009	0.030	0.090
MC-LY	0.006	0.018	0.060	0.180
MC-LW	0.004	0.013	0.040	0.120
MC-LF	0.005	0.016	0.050	0.150

* Limits of detection (LODs) for intracellular toxins altered based on the sample volume that passed through the filter. Limits reported in the table are referred when 150 mL of water sample passed through the filter.

Table S2. Results of untargeted HS-SPME-GC/MS screening per sample.

No.	Sample ID	1	2	3	4	5	6	7	8	9	10	11	12	13	14	15	16
	Sample details / Compound	S1, 22/8/2019	S2, 02/10/2019	S4, 02/10/2019	S1, 22/12/2019	S1, 15/01/2020	S4, 04/02/2020	S5, 15/02/2020	S5, 17/02/2020	S1, 22/02/2020	S2, 22/02/2020	S1, 15/04/2020	S2, 22/04/2020	S4, 22/04/2020	S1, 14/08/2020	S2, 14/08/2020	S3, 09/10/2020
1	α-Thujene											*					*
2	2,2,4,6,6-pentamethyl heptane	*	*	*	*	*	*	*	*	*	*	*	*	*	*	*	*
3	2,2,6-trimethyl-cyclohexanone												*				
4	p-Cresol		*										*				
5	2,4-Dimethyldecane													*			
6	Nonanal		*	*			*			*	*			*	*	*	*
7	4,7-Dimethylundecane																*
8	β-Cyclocitral		*		*												*
9	2,6,6-Trimethyl-1-Cyclohexene-1-acetaldehyde												*				
10	6,10-Dimethyl-5,9-Undecadien-2-one												*				
11	β-Ionone				*								*				*
12	Pentadecane												*				
13	2,4-di-tert-butylphenol	*		*	*		*			*	*			*	*	*	*
14	Hexadecane											*	*				
15	Heptadecane											*	*	*	*	*	*
16	Diisobutyl phtalate			*	*						*		*				
17	Dimethyl disulfide				*												
18	Dimethyl trisulfide				*												
19	Dimethyl tetrasulfide				*												
20	3-methylindole		*		*												

* Identified compounds.

Table S3. LC-MS/MS detection parameters of targeted cyanotoxins (Zervou et al, 2017*).

Cyanotoxin	tR (min)	Precursor ion (*m*/*z*)	Product ion Q1 (*m*/*z*)	Product ion Q2 (*m*/*z*)	Product ion Q3 (*m*/*z*)	[Q2]/[Q1] (%RSD)	[Q3]/[Q1] (%RSD)
CYN	2.10	416.1 $[M+H]^+$	194	336	176	0.39 (1.9)	0.32 (3.0)
ATX	2.96	166.1 $[M+H]^+$	149	131	91	0.72 (2.2)	0.30 (3.4)
[D-Asp3] MC-RR	15.25	512.9 $[M+2H]^{2+}$	135	103	127	0.42 (1.9)	0.34 (2.4)
MC-RR	15.57	519.9 $[M+2H]^{2+}$	135	105	127	0.39 (4.4)	0.35 (5.5)
NOD	17.42	825.3 $[M+H]^+$	135	227	781	0.78 (7.2)	0.13 (13.0)
MC-YR	18.81	1045.2 $[M+H]^+$	213	135	375	1.00 (19.5)	0.62 (24.0)
MC-HtyR	19.29	1059.5 $[M+H]^+$	135	213	375	0.91 (16.3)	0.32 (25.4)
[D-Asp3] MC-LR	19.58	981.6 $[M+H]^+$	213	135	375	0.96 (11.8)	0.77 (14.3)
MC-LR	19.72	995.5 $[M+H]^+$	135	213	105	0.82 (19.5)	0.17 (35.6)
MC-HilR	21.31	1009.6 $[M+H]^+$	135	213	599	0.67 (7.9)	0.43 (22.5)
MC-WR	22.03	1068.5 $[M+H]^+$	213	135	599	0.48 (25.7)	0.35 (47.2)
MC-LA	27.06	910.4 $[M+H]^+$	776	375	402	0.57 (7.7)	0.57 (6.9)
MC-LY	27.84	1002.4 $[M+H]^+$	868	265	494	0.83 (8.7)	0.81 (15.0)
MC-LW	30.15	1025.4 $[M+H]^+$	517	375	446	1.17 (30.6)	0.92 (23.1)
MC-LF	30.67	986.2 $[M+H]^+$	853	478	375	0.95 (16.9)	0.44 (17.6)

* Zervou, S.-K.; Christophoridis, C.; Kaloudis, T.; Triantis, T.M.; Hiskia, A. New SPE-LC MS/MS method for simultaneous determination of multi-class cyanobacterial and algal toxins. *Journal of Hazardous Materials* **2017**, *323*, 56–66, https://doi.org/10.1016/j.jhazmat.2016.07.020.

Table S4. Performance characteristics of the method for the analysis of target MCs in fish flesh and liver.

Fish Flesh	[D-Asp³] MC-RR	MC-RR	MC-YR	MC-HtyR	[D-Asp³] MC-LR	MC-LR	MC-HilR	MC-WR	MC-LA	MC-LY	MC-LW	MC-LF
LOD flesh (ng/g dw)	2.0	1.0	4.0	7.0	4.0	4.0	6.0	6.0	3.0	6.0	4.0	5.0
LOQ flesh (ng/g dw)	6.0	3.0	12.0	21.0	12.0	12.0	18.0	18.0	9.0	18.0	12.0	15.0
% Recovery	80.4	81.6	76.8	45.6	72.5	78.8	68.5	10.5	75.4	76.8	22.2	29.5
intra-day % rsd	7.4	6.8	11.4	11.2	11.2	12.2	16.5	26.5	6.5	5.6	23.4	13.5
**Fish Liver**	**[D-Asp³] MC-RR**	**MC-RR**	**MC-YR**	**MC-HtyR**	**[D-Asp³] MC-LR**	**MC-LR**	**MC-HilR**	**MC-WR**	**MC-LA**	**MC-LY**	**MC-LW**	**MC-LF**
LOD liver (ng/g dw)	1.6	0.8	3.2	5.6	3.2	3.2	4.8	4.8	2.4	4.8	3.2	4.0
LOQ liver (ng/g dw)	4.8	2.4	9.6	16.8	9.6	9.6	14.4	14.4	7.2	14.4	9.6	12.0
% Recovery	65.5	71.8	71.2	39.9	69.5	70.4	61.5	5.5	72.2	70.1	19.2	16.5
intra-day % rsd	12.2	11.1	5.5	10.3	14.4	15.2	15.5	28.4	9.5	12.2	30.2	33.1

Table S5. Physicochemical parameters of Lake Karaoun.

Sampling Date	Aug-19	Sep-19	Feb-20	Mar-20	Apr-20	Aug-20
	Concentration (mg/L)					
Nitrate-nitrogen (NO_3-N)	2.71	0.56	3.39	16.72	6.01	-
Nitrite-nitrogen (NO_2-N)	-	-	0.28	0.06	0.03	0.02
Phosphate-phosphorus (PO_4-P)	-	-	0.10	0.16	0.08	0.04
